# Wild-type and engineered adeno-associated viral vectors produce comparable opsin expression and light-evoked responses in rat skeletal muscle

**DOI:** 10.1016/j.omtm.2025.101559

**Published:** 2025-08-12

**Authors:** Fiona L. Knapman, E. Myfanwy Cohen, Tom Kulaga, Nigel H. Lovell, Leszek Lisowski, Peter G.R. Burke, Lynne E. Bilston

**Affiliations:** 1 Neuroscience Research Australia, Sydney, NSW 2031, Australia; 2 Graduate School of Biomedical Engineering, UNSW, Sydney, NSW 2033, Australia; 3 School of Psychology, UNSW, Sydney, NSW 2033, Australia; 4 Children’s Medical Research Institute, Sydney, NSW 2145, Australia; 5 Military Institute of Medicine – National Research Institute, 04-141 Warsaw, Poland; 6 Macquarie Medical School, Macquarie University, Sydney, NSW 2113, Australia

**Keywords:** musculoskeletal, AAV, optogenetics, muscle stimulation, AAVMYO, AAV9, skeletal muscle

## Abstract

Optogenetics offers a minimally invasive, low-fatigue, and temporally precise alternative to electrical stimulation for skeletal muscle control. After opsin expression in muscle cells, contraction can be stimulated with light. Obstructive sleep apnea, characterized by repeated airway collapse during sleep, suits this approach, as upper airway muscles are readily accessible via the oral cavity, and require stimulation synchronized to respiration. This study compared wild-type (adeno-associated virus 9 [AAV9]) and engineered (AAVMYO) viral vectors for the delivery of identical muscle-specific optogenetic constructs in rats. Three weeks after intramuscular injections, both vectors produced comparable opsin expression in the tongue (*p* = 0.54) and near-zero expression in non-target tissues. AAVMYO-treated animals had greater light-evoked increases in muscle activation than those treated with AAV9 (8.5-fold vs. 2.0-fold; *p* < 0.0001). Conversely, AAV9-treated animals had greater light-evoked airway dilation (2.1 mm^2^ vs. 0.3 mm^2^; *p* = 0.02). By 12 weeks, opsin expression declined to near-zero (vs. 3 weeks; *p* < 0.0001), light stimulation no longer increased muscle activation (*p* > 0.05), and anti-AAV antibodies had significantly increased (*p* < 0.0001). Unlike in mice, AAVMYO did not consistently outperform AAV9 in delivering gene therapy to rat muscles. Despite this, these data support optogenetics for obstructive sleep apnea, although sustained efficacy requires a better understanding of host immune responses and potentially transient immune suppression.

## Graphical abstract

**Figure:**
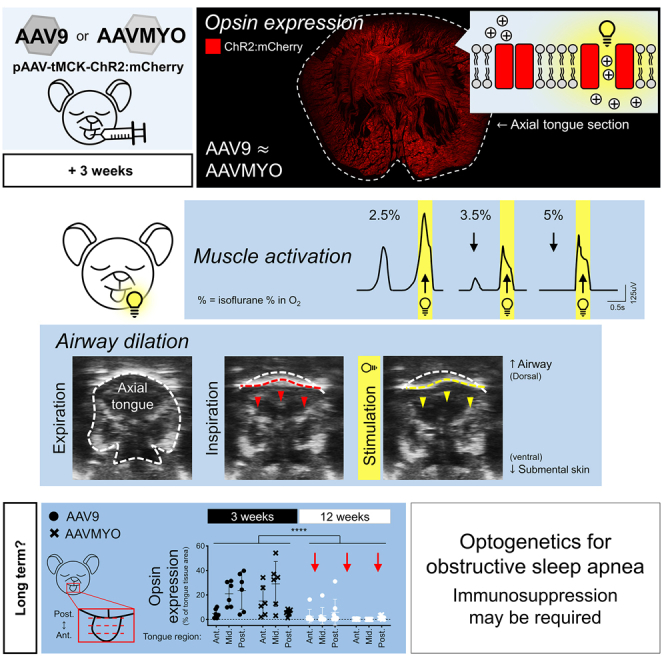

## Teaser

Direct optogenetic stimulation of skeletal muscle offers a promising alternative to nerve stimulation. In this study, wild-type and engineered adeno-associated viral vectors produced equivalent opsin expression in upper airway muscles, enabling light-evoked muscle activation and airway dilation, and supporting the development of a novel therapy for obstructive sleep apnea.

## Introduction

Restoring or enhancing muscle function through the direct stimulation of muscle tissue has therapeutic potential for a range of disorders, from muscular dystrophies to spinal cord injury. Conventional treatments often rely on electrical stimulation of the peripheral nerve, which requires chronic electrode implantation and has several limitations, including poor specificity and rapid muscle fatigue. Optogenetics, which uses light-sensitive proteins to control cellular activity, has emerged as a promising alternative, capable of reinstating functional deficits independent of the underlying defect.^1^^,^^2^^,^^3^ Proof-of-concept studies have demonstrated the feasibility of direct optogenetic activation of skeletal muscle^4^^,^^5^^,^^6^^,^^7^^,^^8^; however, robust and tissue-specific opsin expression in targeted muscles requires an efficient delivery vector. Viral vectors are often used to achieve this, but need to be optimized for best results.

Recombinant adeno-associated viral vectors (rAAVs) are a leading platform for gene delivery. To date, eight rAAV-based therapies have gained regulatory approval from the US Food and Drugs Administration (FDA) and/or the European Medicines Agency. More than 230 clinical trials using AAVs, identified in a 2024 review,^9^ have been completed or are ongoing to investigate their efficacy, safety, and tolerability. In the context of optogenetics, clinical trials for retinitis pigmentosa use rAAVs to drive opsin expression in the retina (e.g., NCT02556736, NCT03326336, NCT04945772, and NCT04278131), and four pre-clinical studies use rAAVs to express opsin in skeletal muscle.^5^^,^^6^^,^^7^^,^^8^ All clinically approved gene therapies, and all pre-clinical optogenetic muscle-stimulation studies use wild-type rAAVs, which have several disadvantages. First, wild-type AAVs are endemic in humans, leading to high rates of pre-existing adaptive immunity among patients.^10^ Since even low antibody titers can eliminate large viral loads^11^^,^^12^ and thus reduce transduction efficacy,^13^ clinical use or enrollment in clinical trials typically requires low or negative antibody titers.^14^^,^^15^^,^^16^ Wild-type rAAVs are also prone to liver sequestration, particularly following systemic administration. This reduces the number of AAV particles available for on-target transduction, necessitates higher viral doses,^17^^,^^18^ and in consequence increases the risk of serious hepatotoxicity.^17^^,^^19^ Higher vector doses not only increase the cost of those potentially life-saving therapies but more importantly increase the risk of harmful immune responses or even death.^19^^,^^20^ Engineered rAAVs may overcome these disadvantages, and there is increasing pre-clinical support for their use.^21^^,^^22^^,^^23^^,^^24^ Rational design, where site-specific modifications are made to existing capsids,^24^^,^^25^ or directed evolution, where libraries of diverse capsids are generated via gene shuffling and/or random mutagenesis,^21^^,^^22^^,^^26^ have produced rAAVs with enhanced tissue specificity, improved liver detargeting,^21^^,^^22^^,^^24^ and/or an ability to evade neutralization by pre-existing anti-AAV-neutralizing antibodies.^23^^,^^26^^,^^27^^,^^28^ Clinically, this has the potential to reduce therapeutic viral doses and to lower the risk of immune responses and serious adverse events.

AAV9, a wild-type serotype, is an efficient option for muscle transduction and is the only serotype used in pre-clinical studies of optogenetics-based muscle stimulation.^5^^,^^6^^,^^7^^,^^8^ The clinical application of AAV9 for pediatric spinal muscular atrophy targets motoneurons rather than skeletal muscle, yet provides evidence of AAV9’s efficacy in humans.^29^^,^^30^ Recently, several engineered serotypes (e.g., the AAVMYO and MyoAAV families) have generated stronger transgene expression in skeletal muscle compared with their parent capsid, AAV9, with lower affinity for other cell types. Systemic administration of AAVMYO in mice generated ∼61-, 17-, and 11-fold greater mRNA levels in the diaphragm, quadriceps femoris, and heart respectively, and ∼9-fold detargeting from the liver.^22^ AAVMYO and other engineered and highly myotropic rAAVs (e.g., AAVMYO2, AAVMYO3,^24^ and MyoAAV^21^) are potentially safer and more effective options for muscle-targeted gene therapies.

To date, rAAV-mediated gene therapies have primarily targeted rare monogenetic disorders but may also benefit more common conditions like obstructive sleep apnea (OSA), a disorder characterized by repeated airway narrowing and collapse during sleep. Current therapies for OSA tend to be poorly tolerated, have variable and unpredictable efficacy, or involve highly invasive procedures.^31^^,^^32^^,^^33^ Hypoglossal nerve stimulation, the only approach that directly targets upper airway muscle dysfunction, is limited to specific patient populations (adult patients with a body mass index of  35 kg/m^2^, moderate-to-severe OSA, predominantly obstructive events, no complete concentric palatal collapses during drug-induced sleep endoscopy, and non-adherence or intolerance to the gold standard therapy, continuous positive airway pressure) and requires the implantation of an impulse generator beneath the clavicle, a respiration-sensing electrode between the external and intercostal muscles, and a stimulating electrode cuffed around the hypoglossal nerve.^34^ An optogenetic-based alternative to peripheral nerve stimulation is particularly attractive for OSA, as the upper airway muscles that fail to maintain airway patency in OSA patients are cyclically and unconsciously recruited in healthy individuals and are readily accessible through the oral cavity. In contrast with indirect activation via the hypoglossal nerve, direct optogenetic activation of the upper airway muscles offers a non-invasive and highly flexible approach to maintaining airway patency, capable of targeting different and/or multiple sites of airway collapse. This study aims to further optimize an rAAV construct for an optogenetics-based muscle stimulation therapy targeting the upper airway muscles, building on the work described in Knapman et al. (2023).^6^ It also marks the first use of AAVMYO in rats, contributing to the growing body of research on viral vectors for muscle gene therapy. The study was designed to assess (1) whether AAVMYO produced stronger opsin expression and light-evoked muscle-activation and airway dilation compared with AAV9; (2) whether expression and viral vector distribution was specific to the targeted muscles; (3) whether functional responses persisted between 3 and 12 weeks post administration; and (4) whether the two serotypes generated different antibody responses. In contrast with most skeletal muscles, where parallel myofibers span two bones to enable movement and thus force generation is the obvious functional metric for stimulation efficacy,^4^^,^^5^ the muscles in the tongue are unique due to their complex interwoven fiber orientations. Moreover, in the context of OSA, airway dilation is a more physiologically and clinically relevant outcome^35^^,^^36^^,^^37^^,^^38^^,^^39^ than force production. As such, stimulation efficacy was assessed by quantifying light-evoked airway dilation under ultrasound imaging.^6^ Light-evoked muscle activation was also evaluated using electromyography (EMG), and opsin expression and vector distribution were evaluated using immunohistochemistry and qPCR, respectively.^40^ Finally, rAAV-specific antibody responses were measured using ELISAs.

## Results

### AAVMYO and AAV9 produced robust expression in the tongue but expression did not persist

All animals tolerated the intramuscular tongue injections well, exhibiting no bleeding or only minor bleeding at the injection site. After recovery from anesthesia, animals promptly resumed normal eating and drinking behaviors and showed no signs of pain or distress. Upon tissue extraction, histological imaging of the tongue revealed no evidence of persistent tissue damage, remodeling or sarcopenia.

**Figure 1:**
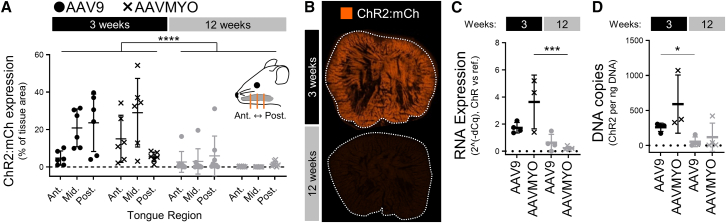
AAVMYO and AAV9 both produced robust expression in the tongue, but neither exhibited persistent expression (A) AAVMYO and AAV9 produced robust and similar opsin:reporter expression in the tongue (LMM, serotype, *p* = 0.54), and similar (serotype × time, *p* = 0.51) and significant reductions in opsin expression between 3 and 12 weeks post rAAV administration (time, *p* < 0.0001), as evident in (B) sample tissue sections at 3 weeks (top) and 12 weeks (bottom). (C) Both serotypes also produced similar RNA expression 3 weeks after administration (Bonferroni multiple comparisons, *p* = 0.23), but only AAVMYO-treated animals then had significant declines in RNA expression (Bonferroni, *p* < 0.001 vs. *p* = 0.06). By 12 weeks, RNA expression was still considered equivalent in both serotype groups (Bonferroni, *p* = 0.06). (D) Three weeks after rAAV administration, vector DNA in the tongue was similar between both serotype groups (Bonferroni, *p* = 0.35). While only the AAV9-treated animals had significant declines in vector DNA between the 3- and 12-week time points (Bonferroni, *p* = 0.007 vs. *p* = 0.13), it was consistent among all animals at 12 weeks (Bonferroni, *p* = 0.28). Data are mean ± SD; ∗∗∗∗*p* < 0.0001, ∗∗∗*p* > 0.001, ∗∗*p* < 0.01.

Opsin:reporter expression, transgene mRNA and vector DNA in the tongue were quantified 3 and 12 weeks post injection using confocal imaging and qPCR, respectively. At 3 weeks, AAV9- and AAVMYO-injected tongues had comparable robust opsin expression (Figure 1A; Bonferroni, *p* = 0.98), transgene mRNA (Figure 1C; Holm, *p* = 0.23), and transgene DNA levels (Figure 1D; Holm, *p* = 0.35). Between 3 and 12 weeks, opsin expression in the tongue declined 7-fold (Figures 1A and 1B) (linear mixed model (LMM): F(1,24) = 32.5, *p* < 0.0001), in all animals, regardless of serotype (LMM: serotype × time, F(1,24) = 0.44, *p* = 0.53). mRNA in the tongue fell 16-fold in AAVMYO-treated animals (Figure 1C; Holm, *p* < 0.001), but only 3-fold in AAV9-treated animals (Holm, *p* = 0.07). Vector DNA copies in the tongue declined 4.9- and 4.7-fold in AAV9- and AAVMYO-treated animals, respectively, but only the former was significant (Figure 1D; Holm, AAV9: *p* = 0.01; AAVMYO: *p* = 0.13). By 12 weeks, opsin expression, RNA expression, and vector DNA were similar regardless of serotype (opsin: Bonferroni, *p* = 0.33; RNA: Holm, *p* = 0.25, DNA: Holm, *p* = 0.28).

As expected following injections targeted to the middle and posterior regions of the tongue, the spatial distribution of opsin expression varied significantly between the anterior, middle and posterior tongue sections (Figure 1A) (LMM: F(1,24) = 56.4, *p* < 0.001). Specifically, the middle section had more than double the opsin expression than the anterior section (Bonferroni, *p* = 0.001), but did not differ from the posterior section (Bonferroni, *p* = 0.12). Distribution did vary between serotype groups (LMM: serotype × section, F(2,48) = 9.4, *p* < 0.001). In AAVMYO-treated animals, the middle section had 4-fold greater opsin expression than the posterior region (Bonferroni, *p* = 0.001), but was not significantly different from the anterior section. In AAV9-treated animals, opsin expression in the middle and posterior sections was 3- and 4-fold greater, respectively, than in the anterior sections (Bonferroni, *p* = 0.01 and *p* = 0.001). Additional main and interaction statistics are provided in the supplemental information (Table S1).

The cohort whose tissue was assessed at 12 weeks post-rAAV administration included male and female animals. When sex was included as a fixed factor, linear mixed model analysis found that opsin expression in the tongue did not differ significantly between the sexes, either across the whole cohort or within the serotype groups (all fixed effects involving sex, e.g., sex and sex × serotype: LMM, *p* > 0.05).

### AAVMYO and AAV9 produced minimal off-target opsin, mRNA, and DNA levels

**Figure 2:**
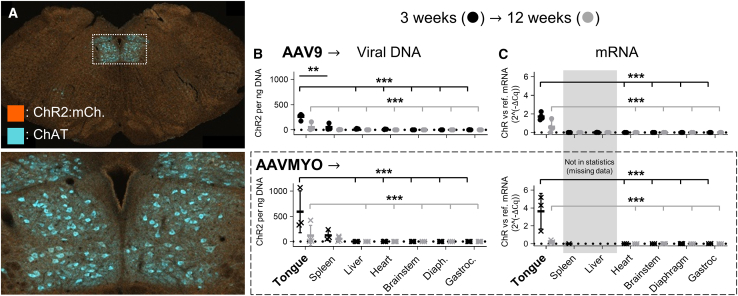
AAVMYO and AAV9 produced similarly low levels of vector DNA and RNA in non-target tissues (A) No animals had opsin:reporter expression in the hypoglossal motor nucleus of the brainstem (ChAT-positive hypoglossal motor neurons and dorsal motor neurons of the vagus are seen (cyan)). (B) At 3 and 12 weeks after rAAV9 administration, vector DNA copy numbers were significantly greater in the tongue compared with all other tissues, with the exception of AAVMYO-treated spleen. (C) At 3 and 12 weeks after rAAV9 administration, vector DNA copy numbers were significantly greater in the tongue compared with all other tissues. Liver and spleen tissues were not included in statistical analysis, since qPCR results indicated undetermined RNA expression in the liver at 3 weeks and in the liver and spleen at 12 weeks following qPCR of AAVMYO-treated tissue. Data are mean ± SD; ∗∗∗*p* < 0.001, ∗∗*p* < 0.01.

To assess biodistribution, confocal imaging was used to examine opsin:reporter protein expression in the hypoglossal nucleus of the brainstem, which could occur following retrograde transport of the rAAV from the tongue if there was expression in the peripheral nerve. Neither AAV9 nor AAVMYO produced detectable opsin expression in the hypoglossal motor nucleus (e.g., Figure 2A). Opsin expression was also absent in related motor nuclei, sensory tracts, and terminal fields, including the facial, trigeminal, and vagal motor nuclei; the sensory trigeminal nuclei; and the nucleus of the solitary tract.

Next, vector DNA and transgene mRNA in various tissues were examined via qPCR. Vector DNA copy numbers were low or undetectable in all off-target tissues (brainstem, diaphragm, gastrocnemius, heart, and liver) both 3 and 12 weeks after AAV9 and AAVMYO administration (Figure 2B, compared with the tongue at 3 and 12 weeks: generalized linear model, Holm, *p* < 0.001), with the exception of the spleen. In AAV9-treated animals, the spleen contained significantly less vector DNA than the tongue at both 3 and 12 weeks post administration (Holm, 3 weeks: *p* < 0.01; 12 weeks: *p* < 0.001). In contrast, following AAVMYO administration, there was no significant difference between in vector DNA levels in the spleen and the tongue at either time point (Holm, 3 weeks: *p* = 0.22; 12 weeks: *p* = 0.29).

mRNA expression was also low or undetectable in off-target tissues, including the brainstem, diaphragm, gastrocnemius, and heart, at both 3 and 12 weeks post AAV9 and AAVMYO administration (Figure 2C, compared with the tongue, Holm, *p* < 0.001). qPCR results showed undetermined mRNA expression in the liver of AAVMYO-treated animals at both time points and in the spleen of AAVMYO-treated animals at 12 weeks. Considering that controls indicated no contamination or unexpected amplification, samples were run in triplicate, and experiments were repeated, it was concluded that the transgene was not expressed at the mRNA level in these tissues. This is supported by the low mRNA expression in the AAV9-treated liver and spleen at 3 and 12 weeks and in the AAVMYO-treated spleen at 3 weeks, which was comparable to other off-target tissues. Due to the undetermined values, the spleen and liver data were not included in statistical analyses.

### AAVMYO-treated animals initially had greater light-evoked muscle activation, but neither group maintained activity at 12 weeks

**Figure 3:**
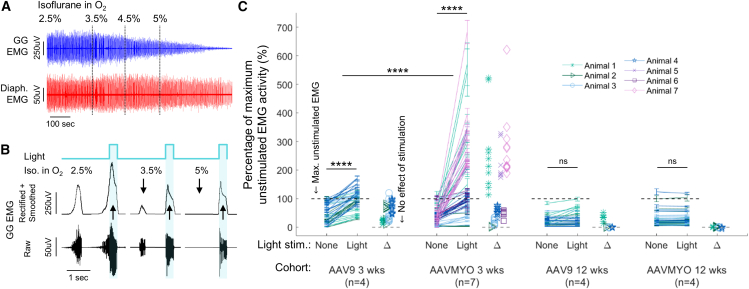
Three weeks after rAAV administration, EMG response to light stimulation was greater in AAVMYO-treated animals than AAV9-treated animals; both groups failed to respond at 12 weeks (A) Increased isoflurane concentration reduces the amplitude of endogenous genioglossus muscle activity (GG EMG) with minimal effects on primary respiratory muscle activation, e.g., the diaphragm (Diaph. EMG). (B) With light stimulation, genioglossus muscle activity increases compared with the prior endogenous (unstimulated) inspiratory muscle activity at all measured endogenous activity levels. (C) Multiple pairs of muscle activation data are plotted per animal (9.9 ± 2.7 pairs of neighboring endogenous and light stimulated EMG). At 3 weeks, tongue EMG significantly increased with direct light stimulation in AAV9- and AAVMYO-treated animals (Bonferroni multiple comparisons, *p* < 0.0001); however, the response was significantly greater in AAVMYO-treated animals (Bonferroni, *p* < 0.0001). At 12 weeks after rAAV administration there was no consistent light-evoked muscle activity in either group (Bonferroni, AAV9: *p* = 0.59, AAVMYO: *p* = 0.96). Data are mean ± SD; ∗∗∗∗*p* < 0.0001.

Three weeks after rAAV administration, all six AAV9-treated animals (100%) and eight of the nine (89%) AAVMYO-treated animals showed increases in tongue EMG in response to direct light stimulation in an isoflurane-based model of upper airway muscle hypotonia and atonia^6^ (Figures 3A and 3B). This was accompanied by light-evoked visible muscle contractions that were greater closer to the LED light source. The induced contractions were visibly larger in the AAVMYO cohort and could be generated across a broad range of LED positions, i.e., when in contact with the lateral, dorsal, or ventral tongue surface. In contrast, the AAV9 cohort required the LED to be in contact with the tongue’s ventral surface. At 12 weeks, only one of four AAV9-treated animals (25%) and none of the four AAVMYO-treated animals (0%) responded to light stimulation with increases in EMG or visible tongue contractions.

EMG data from two animals in the 3-week AAV9 and AAVMYO groups were excluded from quantitative analysis due to heightened isoflurane sensitivity, which abolished inspiratory genioglossus EMG activity with visible muscle contractions at the baseline isoflurane level (2.5%) to which the rest of the data are normalized. All excluded animals had robust light-evoked EMG (e.g., Figure 3B). No animals were excluded from the 12-week data. The response to light stimulation varied between serotype groups and over time (LMM: serotype × time × stimulation, F(1,294) = 27.2, *p* < 0.0001). At 3 weeks, light stimulation increased endogenous muscle activation by ∼8.5-fold in AAVMYO-treated animals (Bonferroni, *p* < 0.0001), and by ∼2-fold in AAV9-treated animals (Bonferroni, *p* < 0.0001). Endogenous muscle activity was comparable between serotype groups (Bonferroni, *p* = 0.44). All light-evoked responses declined over time (LMM: time × stimulation, F(1,293) = 166.1, *p* < 0.0001), and by 12 weeks, light stimulation had no effect in AAV9- or AAVMYO-treated animals (Bonferroni, AAV9: *p* = 0.59, AAVMYO = 0.96). Additional main and interaction statistics are provided in the supplemental information (Table S2).

### Light-evoked airway dilation was strongest in the posterior-axial plane of AAV9-treated animals

**Figure 4:**
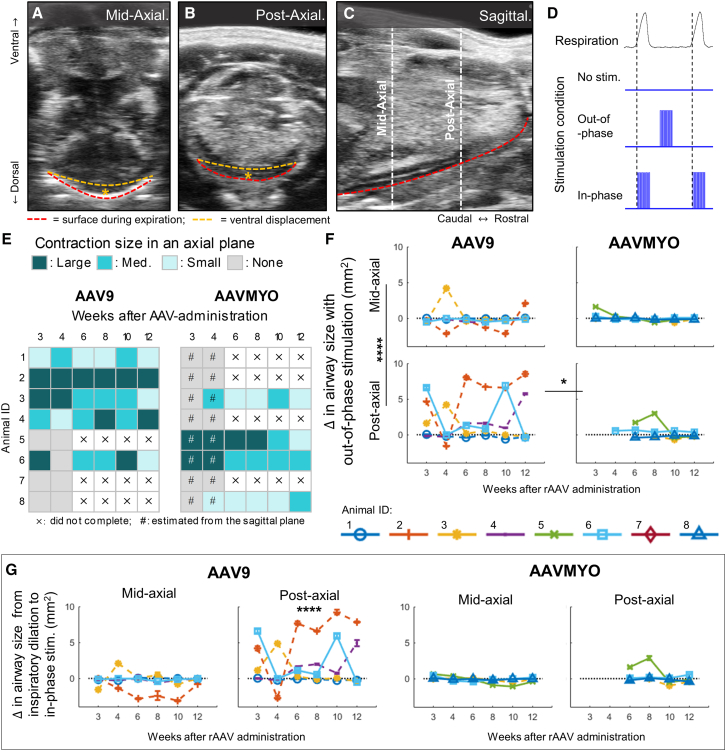
Tongue contraction assessed by ultrasound (A) Tongue contractions were assessed using ultrasound images acquired in the mid-axial plane and (B) the post-axial plane. (C) The locations of the two axial planes are identified in a sagittal view. (D) Ultrasound imaging was performed during normal breathing (no stimulation), out-of-phase stimulation (delivered between breaths, when there is no endogenous tongue movement), and in-phase stimulation (delivered at the onset of inspiration). (E) The area of contracting tissue was estimated as the area of moving tissue in an axial plane when light was applied out of phase with inspiration, and classified as large (>10 mm^2^), medium (>3 mm^2^), or small (<3 mm^2^). In AAVMYO-treated animals, images were not acquired in the post-axial plane at 3 and 4 weeks post-rAAV administration. As such contraction size was estimated from sagittal images (#). (F) Airway dilation due to out-of-phase stimulation was greatest in the posterior axial airway of AAV9-treated animals (Bonferroni, AAV9 mid-vs. post-axial, *p* < 0.0001; post-axial AAV9 vs. AAVMYO, *p* = 0.02). (G) The change in airway dilation between endogenous inspiration and in-phase stimulation was only significant in the posterior axial airway of AAV9-treated animals. Data are mean ± SD; ∗*p* < 0.05, ∗∗∗∗*p* < 0.0001.

Ultrasound imaging quantified upper airway dilation as the displacement of the tongue’s dorsal surface in the axial plane in the middle (mid-axial plane, Figures 4A–4C) and posterior (post-axial plane Figures 4B and 4C) regions of the tongue. The presence of visible muscle contractions was qualitatively recorded as large, moderate, or small, irrespective of their dilatory effect (Figure 4D). Light stimulation was applied out of phase with inspiration (i.e., during expiration so airway dilation was independent of endogenous inspiration-associated dilation) and in-phase with inspiration (i.e., with inspiration onset). The latter was compared with endogenous airway dilation (without stimulation) that had been reduced with isoflurane (Figure 4D).

Light-evoked tongue contractions were visible under ultrasound in five of eight AAV9 animals at 3 and/or 4 weeks post administration (Figure 4E). The remaining three animals had no response at either time point and were excluded from further scans for ethical reasons. Light-evoked tongue contractions were visible in four of eight AAVMYO-treated animals, and the remaining four were excluded from further scans. The data from the non-responders were excluded from statistical analysis.

Light-evoked airway dilation differed in the two axial planes (LMM: axial plane × stimulation, F(2,255) = 10.3, *p* < 0.0001), with out-of-phase stimulation increasing airway size in the post-axial plane by 1.3 ± 0.3 mm^2^, but leaving the mid-axial plane unchanged (mean ± SD) (Figure 4F, Bonferroni, *p* < 0.001). In-phase stimulation increased airway dilation compared with endogenous dilation in the post-axial plane by 1.4 mm^2^ (Figure 4G, Bonferroni, *p* < 0.001), whereas in-phase stimulation did not measurably increase airway dilation in the mid-axial plane compared with endogenous dilation (Bonferroni, *p* = 1.0). In AAV9-treated animals, out-of-phase stimulation increased airway size in the post-axial plane by 2.1 ± 0.4 mm^2^ compared with 0.3 ± 0.6 mm^2^ in AAVMYO-treated animals (mean ± SD) (Figure 4F, Bonferroni, *p* = 0.017). In the mid-axial plane, out-of-phase stimulation produced similar airway dilation in AAV9- and AAVMYO-treated animals (Figure 4F, Bonferroni, *p* = 0.83). In-phase stimulation in AAV9-treated animals increased endogenous inspiratory airway dilation in the post-axial plane by 2.0 ± 0.4 mm^2^ (mean ± SEM) (Figure 4G, Bonferroni, *p* < 0.0001). There was no effect of in-phase stimulation on endogenous airway dilation in either axial plane of AAVMYO-treated animals (Figure 4G, Bonferroni, *p* = 1.0) or in the mid-axial plane of AAV9-treated animals (Figure 3G, Bonferroni, *p* = 0.90). The significantly different light-evoked airway dilation in the post-axial plane between AAV9- and AAVMYO-treated animals may be influenced by the absence of AAVMYO data at 3 and 4 weeks. Posterior-axial images were not initially planned for collection, as the focus was on regions where visible tongue movement had been observed during electrophysiological studies (the posterior airway is not readily visible intraorally). As AAVMYO-treated animals were the first cohort to undergo ultrasound examination, these data are missing. Visual inspection of sagittal images indicated that light-evoked muscle contractions were present in the post-axial plane in these animals (Figure 4E).

All animals with visible light-evoked contractions at the 3- or 4-week scans still produced visible light-evoked contractions at the 12-week time point, albeit at a different amplitude (Figure 4E). This is consistent with the quantitative airway dilation data (Figures 4F and 4G), which found no significant changes in light-evoked airway dilation over the 12-week time period in any group of animals (time stimulation, F(10,225) = 0.11, *p* = 1.0; serotype × time × stimulation, F(10, 225) = 0.31, *p* = 0.98) (additional main and interaction effects are available in the supplemental information, Table S3). However, over the last three ultrasound sessions, it became increasingly difficult to elicit light-evoked airway dilation, often requiring more time to achieve effective LED placement.

Light-evoked muscle contractions within the tongue (Figure 4E) did not always result in airway dilation (Figures 4F and 4G), even when large volumes of tongue muscle moved during contractions. For example, in one animal 4 weeks after AAV9 administration, large visible light-evoked muscle contractions were present (Figure 4E, animal ID 2), but they produced dorsal movement of the tongue dorsal surface, indicating airway narrowing rather than dilation (Figures 4F and 4G, animal ID 2). This was also observed in an AAVMYO-treated animal (animal ID 6, week 4) where large light-evoked tongue contractions (Figure 4E) produced little to no displacement of the tongue’s dorsal surface and therefore no airway dilation (Figures 4F and 4G).

Finally, to assess whether sex influenced the efficacy of light stimulation in producing airway dilation, sex was included as a fixed factor in the linear mixed model analysis of the ultrasound data collected 3 weeks post-rAAV administration. Light-evoked airway dilation was found to be comparable between males and females, regardless of serotype delivered (all fixed effects involving sex and stimulation, e.g., sex × stimulation and sex × stimulation × serotype: LMM, *p* > 0.05). The proportion of animals that did or did not respond to light stimulation also did not differ between the sexes (Fisher’s exact test, *p* > 0.99).

### AAVMYO produced fewer anti-AAV antibodies than AAV9

**Figure 5:**
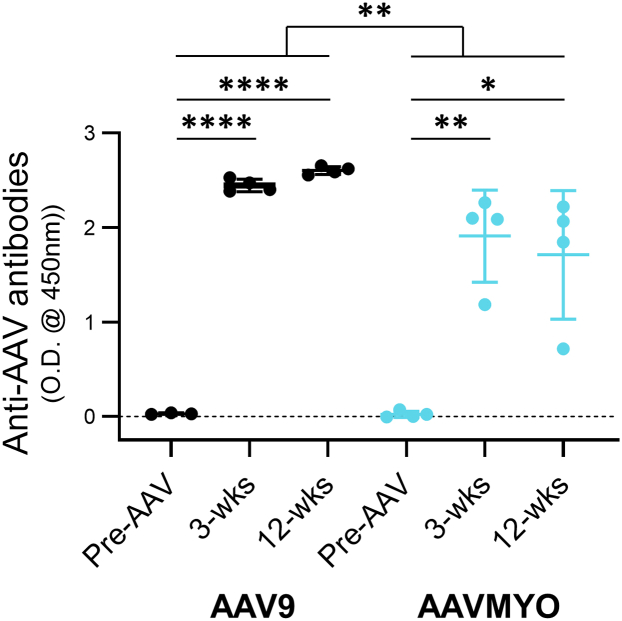
AAVMYO produced fewer anti-AAV antibodies than AAV9 (LMM, Tukey multiple comparisons, *p* < 0.01) Anti-AAV antibodies in plasma significantly increased in all animals by 3 weeks (optical density at 450 nm, AAV9: *p* < 0.0001; AAVMYO: *p* < 0.01) and then persisted to 12 weeks without significant change (AAV9: *p* = 0.65; AAVMYO: *p* = 0.58). Data are mean ± SD, ∗∗∗∗*p* < 0.0001, ∗∗*p* < 0.01, ∗*p* < 0.05.

While no animals had pre-existing anti-AAV antibodies, all had developed them 3 weeks after rAAV administration (Figure 5) (LMM: F(1.34, 11.4) = 87.9, *p* < 0.0001; Tukey, AAV9: *p* < 0.0001, AAVMYO: *p* = 0.009), and they were still present at similar levels at 12 weeks (Tukey, AAV9: *p* = 0.10; AAVMYO, *p* = 0.91). AAV9 generated ∼1.5-fold greater anti-AAV antibodies than AAVMYO across the study period (LMM: F(1,17) = 10.43, *p* = 0.005). However, when each time point was considered separately, there were no significant differences between the cohorts (Tukey multiple comparisons, 3 weeks: *p* = 0.11; 12 weeks: *p* = 0.08).

## Discussion

In an animal model of human sleep-associated upper airway muscle impairment, both AAV9- and AAVMYO generated sufficient opsin expression for robust responses to light stimulation. AAVMYO-treated animals had greater light-evoked muscle activity (EMG), while AAV9-treated animals had greater light-evoked airway dilation measured with ultrasound. Airway dilation was limited to the posterior airway (post-axial plane). As all animals had comparable opsin, RNA, and DNA levels in the tongue 3 weeks after rAAV administration, these discrepancies are likely linked to other factors, such as differences in the distribution of opsins throughout the tongue. Opsin expression and light-evoked muscle activation declined to near-zero levels between the 3 and 12 week time points following rAAV administration. While no reduction in light-evoked airway dilation was recorded, the increased effort required to optimally position the LED does reflect the decline in opsin expression observed in the histology data. The presence of anti-AAV antibodies that had developed in all animals by 3 weeks, and were still present at 12 weeks, may have contributed to these declines. While AAVMYO generated fewer antibodies than AAV9, this did not translate into persistent opsin expression and light-evoked muscle activation. Finally, off-target tissues in all animals had low or absent opsin expression, transgene mRNA expression, and vector DNA, demonstrating that the combination of the muscle-specific promoter tMCK and either AAV9 or AAVMYO capsid resulted in a targeted and restricted expression profile. In sum, both serotypes were equally effective at transducing muscle and offer promising safety profiles for clinical translation.

This study is the first use of AAVMYO in rats, and the first to deliver it via intramuscular injection for local muscle expression. Compared with AAV9, AAVMYO produced similar opsin and nucleic acid levels and distribution and greater light-evoked muscle EMG but smaller light-evoked airway dilation. The first of these is in contrast with the initial AAVMYO publication, which reported 17- to 61-fold greater RNA expression with AAVMYO compared with AAV9 in mouse skeletal muscles following systemic administration.^22^ A subsequent study did reveal strain-dependent variability, with AAVMYO increasing RNA expression in CD1 mice but not in C57BL/6 mice.^41^ Our data suggest that AAVMYO’s efficacy may also be species dependent, and, given that rAAV-mediated gene therapies are currently a one-time treatment with limited potential for re-dosing, this warrants careful consideration for translation to large animal models and human trials. While inter-species variation is an inherent challenge in gene therapies due to factors such as different immune responses,^42^^,^^43^ many rAAVs have demonstrated efficacy across multiple species. MyoAAV, another myotropic engineered serotype derived from AAV9, has driven transgene expression in the skeletal muscle of mice and non-human primates and in human primary myotubes.^21^ AAV9, which allowed light-evoked airway dilation in this study, has driven transgene expression in skeletal muscle of mice,^7^^,^^8^ rats,^6^ canines,^44^ and non-human primates,^45^ and is FDA approved for spinal muscular atrophy, albeit for motor neuron- rather than skeletal muscle-targeted expression.^29^^,^^30^ While engineered rAAVs may eventually surpass AAV9 for systemic skeletal muscle application, AAV9 remains a promising candidate for locally administered viral vector gene therapies targeting skeletal muscle.

### Requirements of an rAAV-mediated muscle gene therapy

Clinical translation of an rAAV-mediated optogenetic muscle-stimulation therapy requires robust long-term opsin expression and consistent light-evoked muscle activity and contraction. In our application (OSA), this is dilation of the upper airway, but the results are applicable to muscle stimulation more broadly. Moreover, stimulation must be achievable during normal physiological behavior, including during body movements or muscle contraction that might cause relative movement of the muscle and the light source. This implies that a system where the stimulation requires very precise positioning of the light source may be impractical clinically. Opsin expression and light-evoked responses must be sustained over time, and the whole therapeutic construct must be safe. This may be best achieved using the minimum effective dose delivered to the target muscles—for OSA, the upper airway dilators—with minimal off-target delivery and expression. Moreover, any immune responses to either the viral vector or transgene need to be clinically manageable to avoid severe adverse events.

#### Robust opsin expression and consistent light-evoked responses

**Figure 6:**
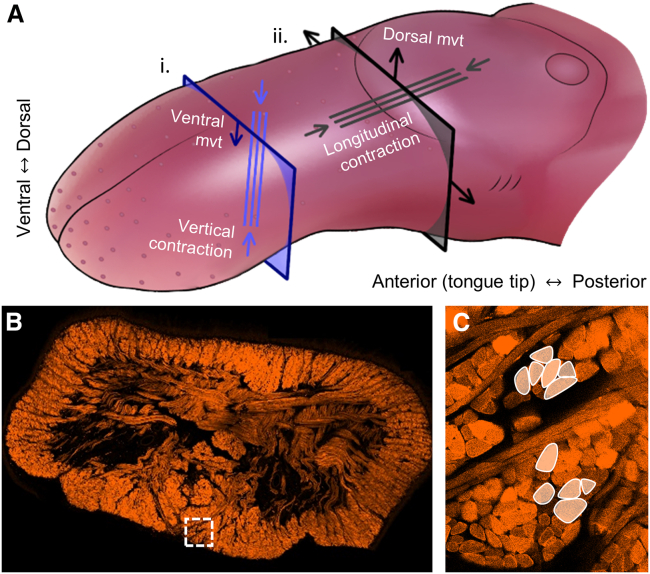
Complex myofiber orientations within the tongue can result in variable deformations detectable in an axial frame (A) (i) Contraction of vertically orientated myofibers (parallel blue lines) produces ventral displacement of the dorsal surface, while (ii) contraction of longitudinally orientated myofibers (parallel gray lines) produces dorsal displacement of the dorsal surface. (B) Mid-axial section of the tongue and (C) highlighted longitudinally oriented myofibers.

Both AAV9 and AAVMYO produced robust opsin expression and light-evoked muscle activation after 3 weeks. However, light-evoked muscle contractions were not always visible under ultrasound examination and did not consistently produce measurable airway dilation. When dilation was recorded, it typically occurred in the posterior axial plane (toward the back of the tongue, Figures 4B and 4C), with only limited effects found more anteriorly (toward the tip of the tongue, Figures 4A–4C). Additionally, the contraction profile was highly dependent on LED position, and small adjustments in LED location altered the pattern of contractions. Several factors may contribute to these observations. First, the expressed opsin, ChR2(H134R), is activated by blue light, which has limited tissue penetration and may not reach deep opsin-positive myofibers. Second, the ultrasound imaging plane was restricted due to obstructions from bony structures (teeth and mandible) and artifacts when the optical fiber enters the transducer’s field of view, which limits light source positions during imaging. In the tongue, due to its complex muscle architecture, slight variations in local activation may produce different movements. Activation of myofibers that are more vertically oriented (e.g., oblique genioglossus, vertical muscles) typically produces airway dilation visible in an axial frame (Figure 6Ai). In contrast, the activation of longitudinally oriented myofibers (parallel to the dorsal surface, e.g., horizontal genioglossus, inferior and superior longitudinal muscles, Figures 6Aii, 6B, and 6C) may draw the posterior tongue anteriorly, resulting in airway dilation visible only in a sagittal frame, but appearing as a visible contraction on ultrasound imaging without dilation in axial frames. As the tongue functions as a muscular hydrostat, longitudinal activation may also cause dorsal displacement of the dorsal surface, resulting in apparent airway narrowing in an axial frame (Figure 6Aii). Moreover, co-activation of the myofibers of different orientations may produce tongue stiffening, with no net displacement of the dorsal surface or airway dilation. The latter has been seen in humans with non-selective electrical stimulation of the tongue.^46^ Finally, the density and distribution of opsin expression throughout the tongue and the position of these opsin-positive myofibers relative to the manually positioned LED during experiments likely contribute to the variation in outcomes of light stimulation in different animals. These factors also likely underpin the lack of consistency between visible tongue muscle movements and airway dilation and the differences between the two serotype groups.

Targeting injections to the dilator muscles, perhaps under ultrasound guidance, could improve the consistency of translating light-evoked muscle activation to light-evoked airway dilation. Indeed, in human hypoglossal nerve stimulation therapies, only dilator muscles innervated by specific branches of the hypoglossal nerve are stimulated, guided in part by the experience of Guilleminault et al.,^46^ who found stimulation of the whole tongue was counterproductive. Red-light-activated opsins, such as ChrimsonR, currently under clinical investigation for retinitis pigmentosa,^47^ could allow the activation of deeper myofibers since red light penetrates muscle better than the blue light used here. That would reduce the need for precise light source positioning and enable transcutaneous submental light stimulation of a greater tongue volume.

#### Persistent opsin expression and light-evoked responses

As re-dosing remains challenging with rAAVs, persistent opsin expression and light-evoked muscle responses following initial rAAV delivery are essential. This was not consistently achieved in the present study. Although some animals continued to respond to light stimulation 12 weeks after rAAV administration, most showed minimal opsin expression and no light-evoked muscle activation or airway dilation. Robust expression at 3 weeks suggests that acute innate immune responses triggered by the injection, or by initial exposure to the rAAV or the opsin did not markedly affect expression. The subsequent decline, coinciding with the appearance of anti-AAV antibodies, likely reflects an adaptive immune response against the capsid, which has been reported in other studies. Opsin-specific immune responses were not captured here and are poorly understood, but may also contribute to declines in transgene expression. Elevated levels of cytotoxic T lymphocytes and macrophages have been documented in sciatic nerve sections containing opsin-positive axons, and ELISAs have found opsin-specific serum antibodies.^48^ In contrast, immune-deficient animals (Rag2^−/−^ rats) and immune-suppressed wild-type animals (slow release tacrolimus, a calcineurin inhibitor, clinically used post organ transplant) had persistent opsin expression and light-evoked responses over 12 weeks.^48^ FDA-approved rAAV-based gene therapies typically require transient immunosuppression, administered prophylactically or reactively, to manage adaptive immune responses and allow persistent transgene expression.^49^ Recent studies also indicate that immunosuppressants can effectively reduce pre-existing antibodies and may enable rAAV administration in individuals with prior rAAV exposure, either through natural infection or previous rAAV-based therapy.^49^^,^^50^^,^^51^ Given the cross-reactivity of anti-AAV antibodies between serotypes, including potential cross-reactivity between antibodies against AAV9 and its descendant serotype, AAVMYO, these interventions may be necessary regardless of serotype used to ensure broader accessibility to therapies. Finally, promoter silencing could contribute to declining opsin expression. However, as viral promoters (e.g., cytomegalovirus) are more prone to silencing compared with non-viral promoters^52^^,^^53^^,^^54^^,^^55^ such as tMCK used here, this mechanism is less likely.

#### AAV9 and AAVMYO provide promising safety profiles with low doses and minimal off-target expression and vector DNA

High vector doses and off-target transgene mRNA and vector DNA distribution are directly related to adverse events in clinical trials of AAV-delivered muscle-directed gene therapies.^17^^,^^19^^,^^56^ With a muscle-specific promoter and intramuscular injections, low doses of AAV9 and AAVMYO are able to produce tongue-specific opsin expression and minimal vector DNA in all other tissues. Vector DNA was slightly elevated in the spleen, potentially due to free rAAV or rAAV-containing immune cells migrating from the tongue via the bloodstream and/or lymphatic system. Prolonged antigen presentation in the spleen may have contributed to the reported increase in anti-AAV antibodies and enabled qPCR detection of vector DNA.

These results contrast with those FDA-approved therapies that systemically administer 27- to 178-fold greater viral loads in order to deliver a transgene to multiple muscles. Systemic delivery, particularly at high viral loads, generates vector DNA sequestration in the liver, increasing the risk of life-threatening hepatotoxicity.^17^^,^^19^^,^^49^ Cross-species validation is needed, but intramuscular delivery of both AAV9- and AAVMYO-packaged constructs show promising safety profiles for eventual clinical translation.

### Implications of optogenetic muscle-stimulation therapy for OSA

In its current form, direct optogenetic activation of the upper airway muscles does not replicate the complex, coordinated patterns of endogenous muscle activity. However, this does not preclude a therapeutic effect. Hypoglossal nerve stimulation offers a precedent; despite producing unilateral contractions in only a subset of tongue dilator muscles, it is able to reduce apneas during sleep. Using ultrasound imaging, we showed that direct light stimulation of the upper airway muscles could produce muscle contractions and/or airway dilation, supporting the concept of an optogenetic muscle-stimulation therapy for OSA. In cases where muscle contractions occurred without measurable airway enlargement, dilation may have occurred in regions not visible because of the imaging plane used, or light stimulation may have generated isometric contractions that increased muscle stiffness in the upper airway, as outlined above. Increasing tissue stiffness through light stimulation may have a protective effect on the upper airway during sleep, even in the absence of airway dilation, since lower tongue stiffness has been linked to greater OSA severity as it lowers the pressure at which the airway collapses.^57^^,^^58^ Future studies could further characterize the effects of light stimulation on the upper airway by incorporating additional axial images and/or using shear wave elastography to quantify muscle stiffness. Quantifying airflow dynamics may also offer additional insight into functional outcomes of light stimulation and aligns with the methodologies used in both pre-clinical and clinical studies of hypoglossal nerve stimulation.

### Conclusions

This study has shown that optogenetic upper airway muscle stimulation can produce upper airway muscle contractions and dilate the airway, showing promise as a therapy for OSA. However further work is needed to improve the consistency of responses, optimize viral loads, and explore transient immune suppression to support long-term efficacy. In this first use of AAVMYO in rats, we found its transduction efficiency to be comparable with that of wild-type AAV9, in contrast with its superior performance in mice.^24^ This suggests species-specific variability in efficacy, highlighting the challenges of translating pre-clinical studies across species.

## Materials and methods

### Animals

All procedures were approved by the Animal Ethics Committees of Macquarie University and UNSW, and accord with The Australian Code of Practice for the Care and Use of Animals. Sprague-Dawley rats were purchased from the Animal Resources Center (Perth, Australia) and were group housed in a dedicated housing room with a 12-h light/12-h dark cycle. Food and water were available *ad libitum*.

### AAV vectors

AAV9- and AAVMYO^22^-packaged constructs were compared. Each contained identical expression cassettes; a muscle-specific promoter (tMCK^59^) and a channelrhodopsin-2 variant with a gain of function substitution (ChR2(H134R)) fused to mCherry (red fluorescent reporter protein), i.e., pAAV-tMCK-ChR2(H134R):mCherry, as described previously.^6^ The AAV9 construct, AAV9-tMCK-ChR2(H134R):mCherry, had a titer of 9.04 × 10^12^ vc/mL, and the AAVMYO construct, AAVMYO-tMCK-ChR2(H134R):mCherry, had a titer of 4.75 × 10^13^ vc/mL. AAV9 animals received 20 μL of the AAV9 construct per animal, equating to 1.81 × 10^11^ vc.^6^ To achieve an equivalent viral load and volume, 3.8 μL of the AAVMYO construct was diluted in 16.2 μL of saline.

The expression cassette, pAAV-tMCK-ChR2(H134R):mCherry, was developed by GenScript (Piscataway, NJ, USA) and was packaged into AAV9 and AAVMYO capsids by the Vector and Genome Engineering Facility (Children’s Medical Research Institute, Westmead, Australia). The AAVMYO plasmid was provided by Dirk Grimm (University of Heidelberg, Heidelberg, Germany).^22^

### Intramuscular AAV injection

Although there is conflicting evidence on the outcome of stimulating both dilator and other muscles in the tongue in humans on airway patency and airflow mechanics,^46^^,^^60^^,^^61^^,^^62^^,^^63^ there may be benefit in co-activating specific regions of the tongue to optimize airway dilation. In this proof-of-concept experiment, we aimed to saturate the tongue body with opsins to deliver a strong signal for comparison between serotypes. Four 5-μL injections of the rAAV packaged construct were administered bilaterally, anterior, and posterior to the lingual frenulum as previously described.^6^

### Study design

Opsin expression (histology), biodistribution (DNA/RNA), light-evoked muscle responses (EMG and ultrasound), and anti-AAV antibody levels were quantified 3 and 12 weeks after rAAV injections. Study stages and cohort numbers are in Table 1. Some data (Table 1) from the AAV9 construct were previously presented in Knapman et al.^6^ Animal weight and age are described in the supplemental information (Table S1).

**Table 1:** Overview of study stages and cohort composition, detailing animal numbers and sex

Study stage			Expression (histology)		Biodistribution (DNA/RNA)		Light-evoked responses				Anti-AAV antibodies		
							EMG		Ultrasound				
Weeks post-rAAV			3	12	3	12	3	12	3	12	pre-	3	12
Serotype (group size and sex)	AAV9	(6M)^a^	✕^a^	–	–	–	✕^a^	–	–	–	–	–	–
		(4M)^a^	–	–	✕^a^	–	–	–	–	–	–	–	–
		(4M)	–	–	–	✕	–	✕	–	–	✕		
		(4M, 4F)	–	✕	–	–	–	–	✕^b^		–	–	–
	AAVMYO	(9M^c^)	6M^c^	–	3M^c^	–	✕	–	–	–	–	–	–
		(4M)	–	–	–	✕	–	✕	–	–	✕		
		(4M, 4F)	–	✕	–	–	–	–	✕^b^		–	–	–

Animals in each cohort completed the stages marked with ✕ in their respective rows.

a Data previously reported in Knapman et al.^6^

b Ultrasound examinations were conducted 3, 4, 6, 8, 10, and 12 weeks after rAAV administration. Animals without a visible light-evoked responses in two consecutive ultrasound examinations did not complete subsequent sessions.

c All animals in this cohort completed the EMG study stage (*n* = 9). Of those, tissues from six animals were used to assess opsin expression, and tissues from three animals were used to assess biodistribution.

#### Opsin expression (histology)

Animals were terminated using intracardiac perfusion with PBS followed by 4% paraformaldehyde. Brain and tongue tissue were extracted and post-fixed with 4% paraformaldehyde overnight. Brainstems were sectioned with a vibratome (80 μm transverse slices, Vibratome VT1200S, Leica, Wetzler, Germany), and stained using primary (tris phosphate-buffered saline with rabbit anti-RFP [Abcam, Cambridge, UK; AB62341], goat anti-Chat [Merck Millipore, Burlington, MA, USA; AB143], and normal donkey serum) and secondary antibody solutions (tris phosphate-buffered saline with donkey anti-rabbit 555 [Invitrogen, Waltham, MA, USA; A32794], donkey anti-goat 647 [Invitrogen A32816], and normal donkey serum) to enhance mCherry fluourescence.^6^^,^^64^ Tongue tissue was sectioned with a cryotome (80-μm coronal slices, Cryostat CM1950, Leica) producing posterior, middle, and anterior sections located approximately 5, 10, and 15 mm from the caudal end of the tongue. All tissue sections were mounted with a fluorescent mounting media (ProLongTM Gold Antifade Mountant, Thermo Fisher Scientific, Waltham, MA, USA) and imaged using a confocal microscope (LSM800 with Airyscan, Zeiss, Jena, Germany). ImageJ was used to manually count mCherry-positive motor nuclei in the brainstem, and to quantify the percentage of tongue tissue above a threshold of no expression.^6^

#### Biodistribution (DNA and RNA quantification)

DNA and RNA distribution were quantified in fresh tissue harvested from animals after saline perfusion.^6^ Tongue, diaphragm, gastrocnemius, heart, liver, spleen, and brainstem were snap-frozen in liquid nitrogen, and minced and divided for separate DNA and RNA extraction. RNA expression was quantified by comparing ChR2 expression with expression of the housekeeping gene YWHAZ. Genomic DNA was assessed with absolute quantification, calculating vector copy numbers per ng of the housekeeping gene RPL13, compared with standard curves that were constructed from linearized plasmid DNA (plasmid 100054, AddGene, Watertown, MA, USA).

#### Light-evoked muscle recruitment (electrophysiology)

Animals were anesthetized in an induction chamber using 5% isoflurane in oxygen.^6^ Isoflurane concentration was reduced to 2.5% via nose cone and then gradually increased by 0.5% increments to a maximum concentration of 5%, reducing and eventually abolishing endogenous tongue muscle activation. This served as a rodent model of human sleep-related upper airway muscle hypotonia and atonia.^6^ EMG data were continuously recorded from two electrodes inserted at approximately the same locations as a unilateral pair of viral vector injections, anterior and posterior to the lingual frenulum. Two diaphragm electrodes were inserted between the caudal intercostal space, and into the costal diaphragm. Light stimulation (470 nm, 10-ms pulses at 40 Hz) was intermittently applied to the ventral and posterior surface of the tongue in phase with inspiration onset. Stimulation was triggered by diaphragm EMG activity exceeding the amplitude of the negative component of the QRS complex of the ECG artifact visible in the signal. To prevent the influence of prior stimulation trains on light-evoked EMG responses, each period of light stimulation was separated by a minimum interval of 30 s.^6^

The effect of light on the genioglossus was quantified by calculating the area under the rectified EMG trace in MATLAB (Natick, MA, USA).^6^ Adjacent unstimulated and stimulated values were calculated by averaging the activity associated with at least five consecutive inspirations. All data were normalized to the maximum unstimulated genioglossus activity obtained at the start of the experiment, when it was visually confirmed that tongue contraction occurred. This approach provides percentages that indicate the degree to which isoflurane reduced unstimulated muscle activity and the extent to which light stimulation was able to restore EMG activity to maximal levels.

#### Light-evoked muscle contractions (high-resolution ultrasound imaging)

Ultrasound imaging was first conducted 3 weeks after rAAV administration. Imaging was then repeated 4, 6, 8, 10, and 12 weeks after rAAV administration. To minimize impact on the animals, subsequent scans ceased when the animal failed to display light-evoked muscle movement at two consecutive scans.

B-mode images were acquired from supine animals (400 planes at 45 planes/second via Vevo3100 and an MX400 ultra-high frequency linear array transducer; FUJIFILM VisualSonics, Ontario, Canada) under three light stimulation conditions and in three anatomical planes.^6^ The three conditions were (Figure 4D) (1) inspiration without stimulation, (2) light stimulation applied out-of-phase inspiration (between expiration and inspiration when endogenous tongue movement was absent), and (3) light stimulation applied in phase with inspiration onset. The three image acquisition planes were (1) a mid-sagittal plane (Figure 4C) positioned between the lingual arteries and extending from the tongue base to where the teeth created an image artifact, (2) a mid-axial plane (Figure 4A), and (3) a posterior-axial plane (post-axial plane, Figure 4B). Images were digitally stored in DICOM format, and quantitative analysis of mid-axial and posterior-axial images was performed in MATLAB. Image registration tracked tongue movement and calculated the change in airway area as defined by the ventral displacement of the tongues dorsal surface during the three stimulation conditions.

#### ELISA measurement of anti-AAV immunoglobulin G-specific antibodies

Blood was collected in EDTA tubes from the saphenous vein prior to rAAV administration and at 3 and 12 weeks after rAAV administration. Plasma was extracted via centrifuge and stored at −80°C until processing. Assays for reactivity to either AAV9 or AAVMYO followed a protocol adapted from Logan et al.^65^ Each well of 96-well polystyrene ELISA plates were coated with 50 μL of each rAAV vector stock diluted to 2.5 × 10^10^ vc/mL in carbonate-bicarbonate buffer (Sigma-Aldrich, St. Louis, MO, USA). Plates were incubated overnight at 4°C. After washing (three washes with PBS + 0.05% Tween 20, Sigma-Aldrich), 100 μL of blocking buffer (PBS + 5% skim milk + 0.05% Tween 20) was added to each well. Plates were incubated for 2 h at room temperature. After washing, 50 μL of plasma (diluted in blocking buffer at 1:50, with duplicate wells for each plasma sample) was added to each well. Plates were incubated for 2 h at room temperature. After washing, 50 μL of horseradish peroxidase-conjugated rabbit anti-rat immunoglobulin G (Sigma-Aldrich AP164) diluted 1:10,000 in blocking buffer was added to each well. Plates were incubated for 1 h at room temperature. After washing, 75 μL of 3,3′,5,5′-tetramethylbenzidine (Sigma-Aldrich) was added to each well. Plates were incubated in the dark for 30 min at room temperature. Reactions were stopped using 75 μL of 1 M H_2_SO_4_ per well. Absorbance of each well at 450-nm wavelength was measured using a microplate reader (PHERAstar FSX, BMG Labtech, Ortenberg, Germany). Duplicate wells containing no AAV served as background controls.

#### Statistics

All data, with the exception of antibody levels, was analyzed in SPSS, and all results are presented as mean ± SD. Opsin expression was analyzed via linear mixed model, using serotype (AAV9 and AAVMYO), tongue section (anterior, middle and posterior), and time (3 and 12 weeks after rAAV administration) as fixed effects, with animal ID as a random effect. Multiple comparisons pairwise comparisons were conducted using the Bonferroni correction.

Biodistribution data (DNA and RNA) were analyzed using a gamma general linear model with variance components. Serotype (AAV9 and AAVMYO), tissue (tongue, brainstem, diaphragm, gastrocnemius, heart, liver, and spleen), and time (3 and 12 weeks after rAAV administration) were considered fixed effects, with animal ID as a random effect. Liver and spleen had undetectable levels of RNA following qPCR, and were not included in the RNA analysis. Multiple comparisons pairwise comparisons were conducted using the Holm correction.

EMG data were analyzed using a linear mixed model. To account for multiple datasets from each animal, adjacent unstimulated and stimulated EMG datasets were grouped based on the unstimulated EMG data remaining following isoflurane induced declines (Figures 3A and 3B). These endogenous groups were created in 10% intervals from 0% to 100% of the maximum unstimulated EMG activity. Endogenous EMG group (1–10), serotype (AAV9 and AAVMYO), time (3 weeks and 12 weeks), and stimulation (stimulated or unstimulated) were used as fixed effects, with animal ID as a random effect. Multiple comparisons pairwise comparisons were conducted using the Bonferroni correction.

Ultrasound data were analyzed using a linear mixed model. Serotype (AAV9 and AAVMYO), stimulation condition (no stimulation, in-phase stimulation, and out-of-phase stimulation), axial plane (middle axial and posterior axial), and time (3, 4, 6, 8, 10, and 12 weeks after rAAV administration) were fixed factors, with animal ID as a random effect. Multiple comparisons pairwise comparisons were conducted using the Bonferroni correction.

Anti-AAV antibody data were analyzed in GraphPad (La Jolla, CA, USA) using a linear mixed model. Time (pre-rAAV administration and 3 and 12 weeks after) and serotype (AAV9 and AAVMYO) were taken as fixed effects, and animal ID was a random effect. Multiple comparisons pairwise comparisons were conducted using a Tukey correction.

## Data availability

The data supporting the findings of this study are included in the article and/or its supplemental information, or are available from the corresponding author (L.B.) upon reasonable request.

## Acknowledgments

We thank Dr. Peter Humburg for statistical assistance and the animal facilities staff at 10.13039/501100001773UNSW Sydney and 10.13039/501100001230Macquarie University for their support with animal care and handling.

We acknowledge the facilities and scientific and technical assistance of the National Imaging Facility, a National Collaborative Research Infrastructure Strategy (NCRIS) capability, at the Biological Resource Imaging Laboratory of UNSW.

This work was funded by an 10.13039/501100000925Australian National Health and Medical Research Council project grant (APP1138808), and a 10.13039/501100001773UNSW Biomedical Engineering seed grant. L.E.B. was supported by an 10.13039/501100000925NHMRC Investigator grant (APP1172988).

## Author contributions

L.E.B. and P.G.R.B. conceptualized the study; T.K. and L.L. provided resources; F.L.K. and E.M.C. performed experiments; F.L.K. analyzed data; F.L.K. and L.E.B. interpreted data; F.L.K. and L.E.B. wrote the initial draft. L.L. and C.M.R.I. assisted with vector construction and troubleshooting. All authors contributed to editing and revising the manuscript and have approved the submitted version of the manuscript.

## Declaration of interests

P.G.R.B. and L.E.B. are inventors on registered patents and patent applications related to optogenetic therapy for OSA.

## Supplemental information

Document S1. Tables S1–S4

Document S2. Article plus supplemental information
