# Supplementary material for: Wild-type and engineered adeno-associated viral vectors produce comparable opsin expression and light-evoked responses in rat skeletal muscle
Source: Mol Ther Methods Clin Dev. 2025 Aug 12;33(3):101559. doi: 10.1016/j.omtm.2025.101559 (PMC12409377; doi:10.1016/j.omtm.2025.101559)
Supplement: Document S1. Tables S1–S4 [file mmc1.pdf]

OMTM, Volume *33*

Supplemental information

Wild-type and engineered adeno-associated viral vectors produce comparable opsin expression and light-evoked responses in rat skeletal muscle

Fiona L. Knapman, E. Myfanwy Cohen, Tom Kulaga, Nigel H. Lovell, Leszek Lisowski, Peter G.R. Burke, and Lynne E. Bilston

Supplemental Material - Statistics

Table S1 | Main and interaction effects of opsin:reporter protein expression in the tongue following a linear mixed model. Variables are animals (inTercept), serotype (AAV9, AAVMYO), time (3- and 12-weeks after viral vector administration) and tongue section (anterior, middle, posterior).

Source of Variation	F (DFn, DFd), P value
Intercept	F(1, 24) = 56.4, p < 0.0001
Serotype	F(1, 24) = 0.4, p = 0.54
Time	F(1, 24) = 32.5, p < 0.0001
Section	F(2, 48) = 7.4, p = 0.002
Serotype × Time	F(1, 24) = 0.4, p = 0.51
Serotype × Section	F(2, 48) = 9.4, p < 0.001
Time × Section	F(2, 48) = 8.5, p < 0.001
Serotype × Time × Section	F(2, 48) = 7.3, p = 0.002

Table S2 | Main and interaction effects of light-evoked electromyography (EMG) following a linear mixed model. Variables are animal (intercept), serotype (AAV9, AAVMYO), time (3- and 12- weeks after viral vector administration), endogenous group (1 to 10 based on the degree of isoflurane induced endogenous muscle activity declines), and light stimulation (stimulated and unstimulated).

Source of Variation	F (DFn, DFd), P value
Intercept	F(1, 17) = 95.1, p < 0.0001
Serotype	F(1, 16) = 3.6, p = 0.08
Time	F(1, 17) = 19.1, p < 0.001
Group	F(9, 297) = 18.1, p < 0.0001
Stimulation	F(1, 293) = 155.6, p < 0.0001
Serotype × Time	F(1, 18) = 2.8, p = 0.11
Serotype × Group	F(9, 297) = 9.1, p < 0.0001
Serotype × Stimulation	F(1, 293) = 55.7, p < 0.0001
Time × Group	F(9, 297) = 10.3, p < 0.0001
Time × Stimulation	F(1, 293) = 166.1, p < 0.0001
Group × Stimulation	F(9, 293) = 11.0, p < 0.0001
Serotype × Time × Group	F(5, 298) = 3.5, p = 0.005
Serotype × Time × Stimulation	F(1, 294) = 27.2, p < 0.0001
Serotype × Group × Stimulation	F(8, 294) = 14.5, p < 0.0001
Time × Group × Stimulation	F(9, 293) = 18.8, p < 0.001
Serotype × Time × Group × Stimulation	F(4, 294) = 9.9, p < 0.001

Table S3 | Main and interaction effects of light-evoked airway dilation under ultrasound imaging following a linear mixed model. Variables are animal (intercept), serotype (AAV9, AAVMYO), time (3- to 12- weeks after viral vector administration), axial section (middle-axial or posterior-axial), and light stimulation (no stimulation, out-of-phase stimulation and in-phase stimulation).

**Source of Variation**	**F (DFn, DFd), P value**
Intercept	F(1, 8) = 6.4, p = 0.04
Serotype	F(1, 8) = 2.1, p = 0.19
Time	F(5, 226) = 0.8, p = 0.54
Axial section	F(1, 226) = 2.6, p = 0.11
Stimulation	F(2, 225) = 2, p = 0.14
Serotype × Time	F(5, 226) = 1.7, p = 0.13
Serotype × Axial section	F(1, 226) = 1.1, p = 0.30
Serotype × Stimulation	F(2, 225) = 1.1, p = 0.35
Time × Axial section	F(5, 226) = 1.6, p = 0.16
Time × Stimulation	F(10, 225) = 0.1, p = 1.00
Axial × Stimulation	F(2, 225) = 10.3, p < 0.0001
Serotype × Time × Axial section	F(4, 226) = 0.4, p = 0.82
Serotype × Time × Stimulation	F(10, 225) = 0.3, p = 0.98
Serotype × Axial × Stimulation	F(2, 225) = 4.4, p = 0.013
Time × Axial × Stimulation	F(10, 225) = 0.3, p = 0.99
Serotype × Time × Section × Stimulation	F(7, 225) = 0.4, p = 0.93

Table S4 | Animal age and weight at rAAV-administration

**Experiment**		**Time**	**Serotype**	**Sex**	**Age**	**Weight**
Expression (histology)		3	AAV9	M	8.1±0.0	256.5±9.7
			AAVMYO		7.7±1.3	321.0±4.7
		12	AAV9	F	6.7±0.0	150.0±5.9
				M	8.0±0.0	333.5±10.5
			AAVMYO	F	7.0±0.0	179.5±8.0
				M	7.0±0.0	251.8±3.3
Biodistribution (DNA/RNA)		3	AAV9	M	7.9±0.0	327.5±9.8
			AAVMYO		6.4±0.0	326.7±9.8
		12	AAV9	M	8.2±0.0	317.8±1.5
			AAVMYO		8.2±0.0	320.8±7.1
Light-evoked responses	EMG	3	AAV9	M	8.1±0.0	256.5±9.7
			AAVMYO		7.2±1.2	322.9±7.4
		12	AAV9	M	8.2±0.0	317.8±1.5
			AAVMYO		8.2±0.0	320.8±7.1
	Ultrasound	All	AAV9	F	6.7±0.0	150.0±5.9
				M	8.0±0.0	333.5±10.5
			AAVMYO	F	7.0±0.0	179.5±8.0
				M	7.0±0.0	251.8±3.3
Anti-AAV antibodies		All	AAV9	M	8.2±0.0	320.8±7.1
			AAVMYO		8.2±0.0	317.8±1.5
